# The Impact of Comorbid Sleep-Disordered Breathing on Hospitalization Risk Related to Diabetes and Atherosclerotic Disease: A Retrospective Cohort Analysis

**DOI:** 10.3390/jcm13247715

**Published:** 2024-12-18

**Authors:** Hlynur Davíð Hlynsson, Jason C. Ong, Joseph Day, Thomas Kauss, Kristófer Montazeri, Jeffrey Hertzberg, Emerson Wickwire, Rebecca M. Hankla, Eysteinn Finnsson, Jón Skírnir Ágústsson, Heidi Riney

**Affiliations:** 1 Nox Health, Inc., 100 Kimball Place, Suite 100, Alpharetta, GA 30009, USA; hlynurd@gmail.com (H.D.H.); rhankla@noxhealth.com (R.M.H.); eysteinnf@noxmedical.com (E.F.); jons@noxmedical.com (J.S.Á.);; 2 Medformatics, Inc., Minneapolis, MN 55419, USA; 3 Division of Pulmonary, Critical Care, and Sleep Medicine, Department of Medicine, University of Maryland School of Medicine, Baltimore, MD 21201, USA; 4 Department of Psychiatry, University of Maryland School of Medicine, Baltimore, MD 21201, USA

**Keywords:** sleep apnea syndromes, diabetes mellitus, hospitalization, cardiovascular diseases

## Abstract

**Objective:** To determine the relationship between comorbid sleep-disordered breathing (SDB) and hospitalization rates related to diabetes mellitus (DM) and atherosclerotic disease (AD). **Methods:** This study used a retrospective cohort design from a large medical claims database with 5 years of data between 2018 and 2022. The presences of SDB, DM, and AD were identified using International Classification of Diseases (ICD-10) and relevant Current Procedural Terminology (CPT) codes. Hospitalizations related to DM and AD were identified primarily using Place of Service (POS) code 21. Propensity-score matching was first used on data from the entire 5-year period to select matched controls (unadjusted n = 883,910, adjusted n = 888,619) compared to those diagnosed with SDB (n = 519,818) on hospitalization rates during the concurrent 5-year period. A second analysis used propensity-score matching on data from year 1 only to select matched controls (unadjusted n = 248,848, adjusted n = 260,298) compared to those diagnosed with SDB in year 1 (n = 193,671) on hospitalization outcomes in the subsequent 4-year period. **Results**: Odds ratios (ORs) revealed a significant association between SDB diagnosis and hospitalizations related to DM (OR 1.23–1.71), AD (OR: 1.08–1.34), and either condition (OR 1.17–1.49) in both analyses. Post hoc analysis revealed sex differences in the relationship between SDB and future hospitalizations, with females showing a pattern of significantly elevated risk across all future hospitalization outcomes (OR: 1.25–1.44), whereas males were found to have a significant relationship between SDB diagnosis and future DM hospitalization only (OR 1.10). **Conclusions:** These findings provide real-world evidence that comorbid SDB increases the risk for hospitalizations related to chronic cardiometabolic conditions. Sex is a potential moderator of this relationship and should be further explored.

## 1. Introduction

Sleep-disordered breathing (SDB) refers to a group of sleep disorders characterized by abnormal and repeated breathing interruptions during sleep, including central and obstructive sleep apnea (OSA). OSA is the most common SDB and is associated with several other chronic diseases, particularly cardiometabolic diseases such as diabetes mellitus (DM) and atherosclerotic disease (AD) [1,2]. Studies have found that 83% of type 2 diabetes patients suffer from undiagnosed sleep apnea [3,4,5]. Notably, individuals with OSA have a heightened risk of DM onset compared to those without the condition [2,4,6]. Studies examining sex differences in DM-associated complications have shown that females with DM have a significantly higher risk of all-cause mortality, hospitalizations, and cardiovascular disease (CVD) compared to males with DM [7]. OSA is also associated with atherosclerotic diseases (ADs), which include cerebrovascular disease, ischemic stroke, coronary artery disease, and peripheral vascular disease. Intriguingly, atherosclerosis manifests in OSA patients even in the absence of other pronounced risk factors [8]. It has been posited that the pathophysiological mechanisms underlying these associations involve oxidative stress and systemic inflammation resulting from recurrent intermittent hypoxia in SDB [9].

While several studies have established a positive association between SDB and an increased prevalence of DM and AD, there is a paucity of real-world data examining the consequences of these comorbidities on health care resource utilization (HCRU). Specifically, hospitalization—a serious and expensive HCRU outcome—is a frequent metric used to indicate worsening disease progression [10,11,12] and is of direct relevance to key stakeholders including patients, payers, and health systems leaders [11,13]. Examining SDB in the context of hospitalization rates related to these chronic conditions can provide important insights into the economic impact of SDB in real-world situations. 

A notable challenge in conducting real-world HCRU research is the difficulty in using rigorous designs that are needed to determine the nature of the relationship between factors and outcomes. Kraemer and colleagues [14] proposed a typology for characterizing the relationship as follows: (1) a *correlate*, if the factor is associated with the outcome; (2) a *risk factor*, if the factor precedes the outcome; (3) a *modifiable risk factor,* if the factor can be changed; and (4) a *causal risk factor*, if manipulation of the factor changes the outcome. While randomized controlled trials remain the gold-standard method to determine a causal risk factor, the methods used to determine a correlate and risk factor are more equivocal and typically involve a process of elucidation [14]. Quasi-experimental designs using statistical procedures for reducing the influence of potential confounders or design strategies to examine temporal relationships between the factor and outcome can serve as appropriate alternatives for assessing long-term outcomes in real-world patients [15].

The purpose of the present study was to investigate the relationship between SDB diagnosis and hospitalization rates related to DM and AD using a large claims dataset. Our hypothesis was that the findings would support the diagnosis of SDB as a risk factor for hospitalizations related to DM, AD, or either. We also explored patterns in the hospitalization rates of males and females separately to generate hypotheses about sex as a potential moderating factor. 

## 2. Materials and Methods

### 2.1. Study Design and Data Source

This study employed a retrospective cohort design using the Merative MarketScan Research Commercial Database (Ann Arbor, MI, USA) between 2018 and 2022. The database was constructed from privately insured, adjudicated medical and prescription drug claims and contains detailed patient-level information, including demographics, medical history, and clinical outcomes. The database was deidentified and HIPAA-compliant and the study was determined to be exempt by the WCG Institutional Review Board under 45 CFR § 46.104(d)(4).

### 2.2. Study Population

**Figure 1:**
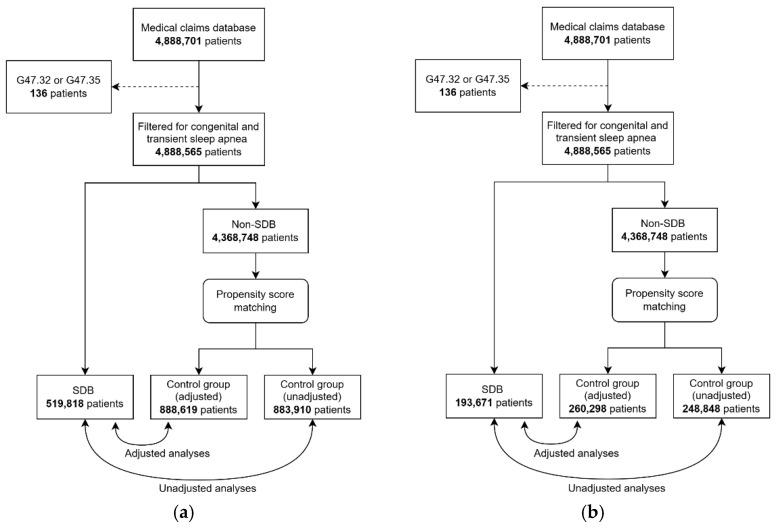
Cohort selection for analyses. Flowchart of cohort selection for each analysis. Panel (**a**) depicts the cohort selection for analysis 1 (5-year matching). Panel (**b**) depicts the cohort selection for analysis 2 (year 1 matching). In each analysis, the SDB group is compared to the control group (adjusted) in the adjusted model and then compared to the control group (unadjusted) in the unadjusted model. G47.32 = high-altitude periodic breathing, and G47.35 = congenital central alveolar hypoventilation syndrome.

The overall database included individuals who were at least 18 years of age as of 1 January 2018 and less than 65 years of age as of 31 December 2022. Additionally, only those who were continuously enrolled during these dates were included. For this study, we excluded patients (n = 136) with ICD-10 diagnostic codes of transient sleep apnea, G47.32 (high-altitude periodic breathing), and sleep apnea identified at birth, G47.35 (congenital central alveolar hypoventilation syndrome). This resulted in a pool of 4,888,565 patients available for selection into the analyses (see Figure 1).

### 2.3. Identification of Chronic Conditions

The chronic medical conditions of interest were identified using a classification schema based on the relevant ICD-10 diagnostic codes, CPT Procedure Codes, and any disease-identifying medications (see Supplementary Table S1). Identification of SDB was based on ICD-10 code G47.3 and CPT code 94660. Identification of DM included a number of ICD-10 codes and CPT codes to capture the various types of diabetes, including Type 1 DM, Type 2 DM, and gestational diabetes. AD included a number of conditions: cardiac dysrhythmias, cerebrovascular diseases (e.g., hemorrhagic or ischemic stroke), congestive heart failure (CHF), coronary artery disease (CAD), peripheral vascular disease (PVD), and kidney diseases. Identification of AD was based on the same classification schema applied to each of these conditions. A similar schema was used to identify other medical conditions used in the matching procedure. 

### 2.4. HCRU Outcomes

The HCRU outcome of interest was hospitalizations related to AD or DM. Hospitalizations were identified using Place of Service (POS) code 21, which encompasses all forms of hospital care excluding psychiatric services. This code accounts for diagnostic, therapeutic (both surgical and nonsurgical), and rehabilitation services provided by, or under, medical supervision. The reason for hospitalization was determined by identifying all claims clustered around a claim with a Place of Service (POS) equal to 21, as documented in the CMS Medicare standards. All overlapping claims were then gathered into a single event, and the claims were sorted by claim amounts in descending order. The largest dollar claims were assigned as the hospitalization condition or reason. Based on this method, any individuals with hospitalizations that were primarily related to AD, DM, or either (AD or DM) during the specified time period were considered positive for the respective outcome measure.

## 3. Data Analyses

### 3.1. Matching Procedure

Propensity-score matching was used to select controls to match SDB patients, ensuring balance between the groups by equalizing the distribution of confounding variables [16]. Although there are limitations to the assumptions of this method, it can serve as a practical and cost-efficient approach to analyze large, generalizable cohorts such as a claims dataset. A logistic regression model was applied to a subset of the data to estimate the propensity scores for each individual, which represents the predicted probability of having a diagnosis of SDB. The variables entered into the model included age, sex, and medical conditions listed in Table 1. We compared the effects of including (adjusted models) or excluding (unadjusted models) a history of AD and DM as features to the propensity score during matching (see analytic plan). Subsequently, the K-nearest neighbors (KNN) algorithm was employed to identify the closest matches based on the propensity scores. Duplicates were removed after matching to ensure that each control patient was unique. To assess the balance between the SDB group and the corresponding control group used for the analysis (adjusted or unadjusted), standardized mean differences (SMDs) were calculated to compare the means of each variable between the two groups, considering the variability within each group [17]. Multicollinearity was considered during the analysis and was found to not influence the propensity-score matching. 

### 3.2. Analytic Plan

Following Kraemer et al.’s [14] recommendations for a sequential process of investigating potential risk factors, we conducted two sets of analyses. The first set was designed to optimize the propensity matching by using data across all five years of the dataset (see Figure 1a). In this analysis, the SDB group (n = 519,818) was selected using the classification schema described above at any point during the 5-year dataset. Control groups were selected using propensity matching from those who did not have an SDB diagnosis in any of the five years (n = 4,368,748) to compare with the SDB group in two models. In the first model (unadjusted model), an unadjusted control group (n = 883,910) excluding DM and AD in the propensity-score matching was used as a reference to compare with the SDB group on hospitalizations during the 5-year period. In the second model (adjusted model), an adjusted control group (n = 888,619) including DM and AD in the propensity score matching was used as a reference to compare with the SDB group on hospitalizations during the 5-year period. The goal of the first analysis (5-year matching) was to determine if SDB diagnosis is a significant correlate of hospitalizations related to DM, AD, or either (DM or AD) in the present sample.

In the second set of analysis, we redesigned the time period for the group selection and the observation period for hospitalization to account for temporal precedence in the relationship between SDB diagnosis and hospitalizations (see Figure 1b). In this analysis, the SDB group (n = 193,671) was selected only from the first year (2018) using the same classification schema described above. Separate control groups were selected from those with no SDB diagnosis in any of the five years (n = 4,368,748), but the propensity matching was conducted using year 1 (2018) data only. Similar to the first set of analyses, two models were used to compare the SDB group to an unadjusted control group (n = 248,848) and an adjusted control group (n = 260,298). However, the observation period for hospitalizations related to DM, AD, or either (DM or AD) was in the subsequent four years (2019–2022) to ensure that all hospitalizations occurred after the SDB diagnosis. The goal of the second analysis (year 1 matching) was to determine if SDB diagnosis is a risk factor for future hospitalizations related to DM, AD, or either (DM or AD). 

For all models, we conducted post hoc analyses with males and females separately to explore patterns for each sex. The significance between SDB and hospitalization outcomes was evaluated by calculating odds ratios with bootstrap sampling using a 95% confidence interval (CI).

## 4. Results

### 4.1. Patient Characteristics

Table 1 presents the demographic and clinical characteristics of the participants in the SDB group and the corresponding control group in each model (adjusted and unadjusted model) for each of the analyses. SMD values were within the recommended range (−0.10 to 0.10) across all variables (age, sex, medical conditions) used for the propensity matching in the 5-year analysis except for the DM or AD variable (SMD = 0.11), indicating that both the unadjusted and adjusted models were effectively balanced [17]. In the year 1 analysis, the SMD exceeded the recommended range on sex in the unadjusted (SMD = −0.25) and adjusted model (SMD = −0.23), with fewer females in each of the respective control groups. This supports the need to conduct post hoc analyses by sex. With regard to medical comorbidities, the SMD was also slightly outside the recommended range on respiratory diseases (SMD = −0.11) in the unadjusted group and on DM (SMD = 0.13) and DM/AD (SMD = 0.11) in the adjusted group. With the exception of these variables, the groups were well balanced in the year 1 analysis. Although the SMD values for DM and AD were outside of the recommended range on the unadjusted models, this was expected because these variables were excluded in the propensity-score matching in these models. 

### 4.2. Analysis 1: 5-Year Matching

**Figure 2:**
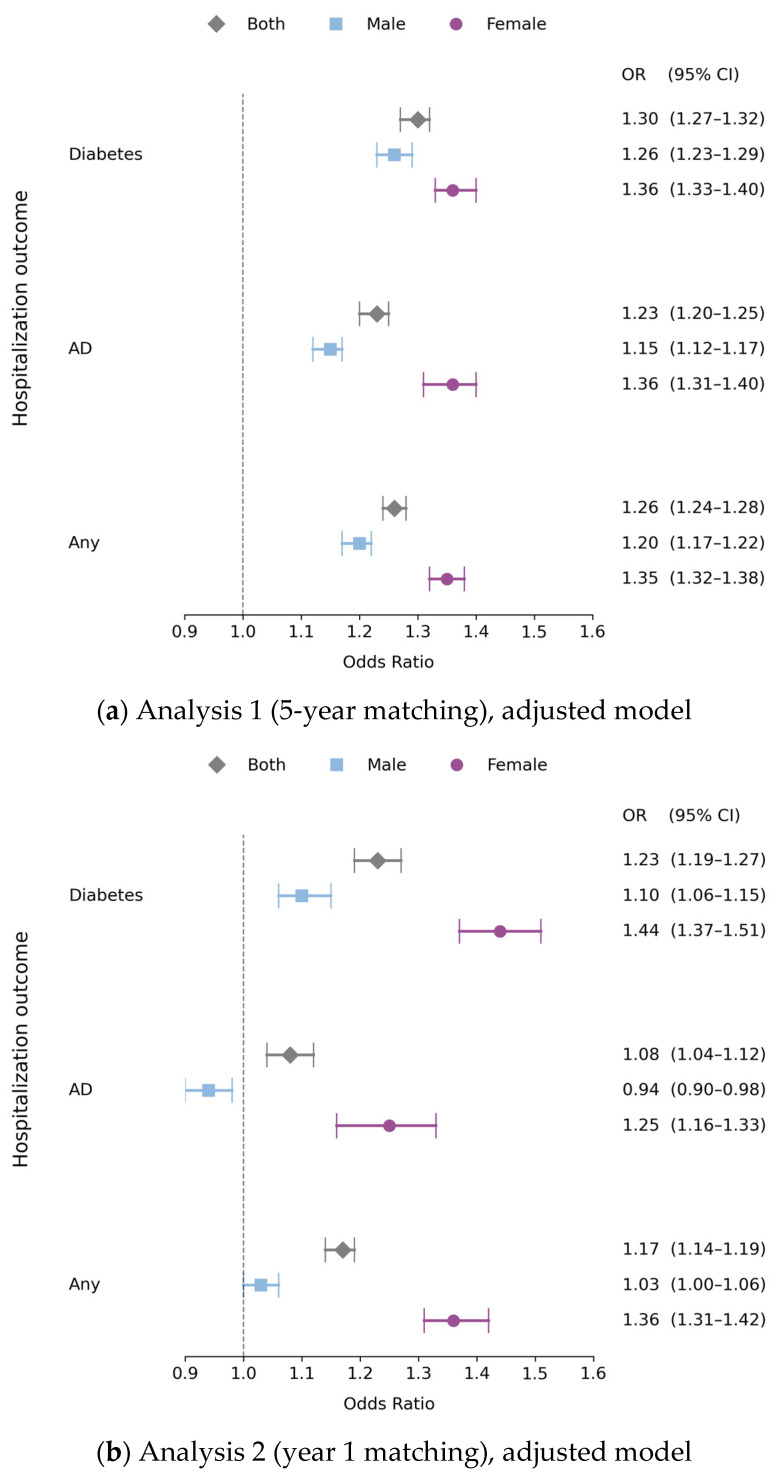
Odds ratios of hospitalization rates for SDB and matched controls. Figure 2 illustrates the odds ratios for hospitalizations due to DM and AD for males, females, and combined groups in the adjusted model in Analysis 1 (**a**) and in the adjusted model in Analysis 2 (**b**). There are notable differences in the odds ratios between males and females for both DM and AD hospitalizations, particularly in Analysis 2. OR = odds ratio, CI = confidence interval.

Using propensity-score matching on all five years of data, both the unadjusted and adjusted models revealed significant associations between SDB diagnosis and hospitalization rates relative to controls for all outcomes (see Table 2). Relative to the unadjusted control group, the odds ratio for the SDB group was 1.63 (95% CI: 1.61–1.67) for DM-related hospitalizations, 1.34 (95% CI: 1.32–1.37), for AD-related hospitalizations, and 1.49 (95% CI: 1.47–1.51) for hospitalization due to DM or AD. Relative to the adjusted control group, the odds ratio for the SDB group was 1.30 (95% CI: 1.27–1.32) for DM-related hospitalizations, the odds ratio was 1.23 (95% CI: 1.20–1.25) for AD-related hospitalizations, and it was 1.26 (95% CI: 1.24–1.28) for hospitalization due to DM or AD. Post hoc analysis revealed a similar pattern of significant associations on all hospitalization outcomes in both males and females with elevated odds ratios for females relative to males (see Table 3 and Figure 2a). 

### 4.3. Analysis 2: Year 1 Matching

Since Analysis 1 established SDB diagnosis as a significant correlate for each of the three outcome variables, Analysis 2 examined if it is a risk factor by accounting for the temporal relationship between SDB diagnosis and hospitalization (see Table 2). Both the unadjusted and adjusted models revealed significant associations between SDB diagnosis and hospitalization rates relative to controls on all outcomes. Relative to the unadjusted control group, the odds ratio for the SDB group was 1.71 (95% CI: 1.65–1.76) for DM-related hospitalizations, 1.17 (95% CI: 1.13–1.22), for AD-related hospitalizations, and 1.45 (95% CI: 1.42–1.49) for hospitalizations due to DM or AD. Post hoc analysis (see Table 3 and Figure 2b) revealed a similar pattern for females, with significant odds ratios for each of the outcome variables. However, for males, the odds ratio was significant for DM-related and DM- or AD-related hospitalizations, but not for AD-related hospitalizations (OR: 1.00. 95% CI: 0.96–1.05). Relative to the adjusted control group, the odds ratio for the SDB group was 1.23 (95% CI: 1.19–1.27) for DM-related hospitalizations, 1.08 (95% CI: 1.04–1.12) for AD-related hospitalizations, and 1.17 (95% CI: 1.14–1.19) for hospitalization due to DM or AD (see Table 2). Post hoc analysis (see Table 3 and Figure 2b) revealed a similar pattern for females, with significant odds ratios for each of the outcome variables, but for males, the odds ratio was only significant for DM-related hospitalizations (OR: 1.10; 95% CI: 1.06–1.15) and not for AD-related (OR: 0.94; 95% CI: 0.90–0.98) or either DM- or AD-related hospitalizations (OR: 1.03; 95% CI: 1.00–1.06). 

## 5. Discussion

The present findings provide general support for the hypothesis that a comorbid SDB diagnosis is a risk factor for hospitalizations related to chronic cardiometabolic conditions. Specifically, SDB was associated with a 30% increased risk of DM-related hospitalizations and a 23% increased risk of AD-related hospitalizations relative to matched controls during the 5-year study period. Analyses accounting for temporal precedence revealed that an SDB diagnosis in the first year was associated with a 23% increased risk of future DM-related hospitalizations and an 8% increased risk of future AD-related hospitalizations in the following four years relative to matched controls. Interestingly, sex differences were found in the relationship between SDB and future hospitalizations, with females having elevated risk across all hospitalization outcomes, whereas males were found to have a significant relationship between SDB diagnosis and future DM hospitalization only. Taken together, the present study provides new insights into the relationship between SDB, sex, and hospitalizations. 

This study adds an important real-world perspective to the growing body of literature indicating the HCRU cost of SDB among adults with chronic cardiometabolic conditions. Although the associations between sleep apnea and DM and AD are well established [4,18], relatively few studies have examined the impact of comorbid SDB on hospitalization. One recent study by Kirk and colleagues [19] found that occult, undiagnosed sleep apnea was associated with increased risk for hospitalization as well as 30-day readmissions among older adult Medicare beneficiaries with pre-existing cardiovascular disease (N = 142,983). The present analysis builds upon and expands these findings by including adults between 18 and 65 years old and also examining the effects of comorbid SDB among individuals with DM. Notably, the association between SDB and DM-related hospitalization was significant across all models (unadjusted and adjusted) and for both sexes, indicating a robust relationship. This finding is consistent with previous research showing that the presence of sleep apnea was associated with higher future HCRU among patients with diabetes undergoing elective surgery [20]. Furthermore, post hoc analyses of sex revealed that among females, SDB diagnosis was consistently associated with a greater risk of future hospitalizations related to DM and AD. In contrast, the relationship was only significant for DM among males. These findings build upon previous work by Roche and Wang [7] showing sex differences in hospitalizations among patients with diabetes by including SDB as another potential factor. One hypothesis for this observation is that women are not receiving adequate treatment for sleep apnea, leading to increased hospitalizations. An alternative hypothesis is that women are more likely than men to experience shorter respiratory events during sleep, but with an elevated heart rate response at the end of these events, which is associated with an increased risk of cardiovascular diseases [21].

Our study design also revealed some noteworthy methodological considerations for using propensity-score matching to analyze large datasets. The analysis using 5-year matching was designed to optimize the propensity matching but did not account for the temporal relationship between SDB diagnosis and hospitalization. Consequently, the results could only determine that SDB was a correlate of hospitalization, which was important to first establish in this sample. Subsequently, the analysis using year 1 matching was designed to elucidate the temporal precedence of the SDB diagnosis occurring prior to hospitalizations, thus supporting the conditions for SDB diagnosis as a risk factor for future hospitalizations. However, the propensity-score matching was limited by having only one year of data, resulting in a suboptimal balance between the apnea and control groups on two variables (sex and respiratory diseases). The post hoc analysis provided insights on sex differences in the risk of future hospitalizations among females, but it is also possible that respiratory conditions other than SDB contribute to increased risk of hospitalizations related to DM and AD. Therefore, we encourage future work in this area to carefully consider the priority and sequence of these issues in relation to the goals of this study and the state of the literature. 

### 5.1. Limitations

While this study offers valuable real-world data, several limitations should be acknowledged when interpreting these findings. Medical claims data do not account for disease severity and could be susceptible to coding inaccuracies or incomplete documentation, impacting diagnostic and hospitalization record precision (e.g., distinguishing OSA and complex sleep apnea). While a diagnosis of SDB can serve as a proxy for these conditions, it is not reliable for determining disease onset since there could be factors other than the onset of symptoms (e.g., presence of an adverse event, treatment-seeking behavior) that determine when a patient decides to undergo evaluation for SDB. This limited our ability to examine the temporal relationship between the onset and progression of SDB relative to the onset and progression of other comorbid conditions. Potential confounding factors that are not measured, such as physical activity and health literacy, could significantly affect DM [22,23] and AD [24,25] risk. Important variables such as sleep apnea treatment among diagnosed patients were not analyzed in this study. The risk of hospitalization could be diminished if patients were already receiving effective treatments such as continuous positive airway pressure (CPAP) therapy. Conversely, the control groups might include undiagnosed SDB individuals, potentially leading to underestimating the association between SDB and hospitalization rates for DM and AD. Either case would cause our effect sizes to underestimate the actual values. Finally, we could not verify if data for sex were assigned at birth or based on gender identity, which could have implications for interpreting the post hoc analyses on sex. 

### 5.2. Future Directions and Conclusions

Future research can add to the growing evidence of the association between SDB and chronic cardiometabolic conditions and the impact on HCRU in several ways. One goal of this study was to lay the foundation for further research aimed at determining if SDB or specific types of sleep apnea can be a *causal risk factor* for hospitalizations and other costly HCRU. This would have important public health and economic implications since sleep apnea is treatable and effective treatment should correspond to a decrease in hospitalizations and HCRU. Peker et al. [26] found that treatment of sleep apnea with positive airway pressure (PAP) can reduce hospitalization due to cardiovascular and pulmonary diseases, while Sterling and colleagues [27] found that higher adherence to PAP is associated with fewer all-cause hospitalizations and emergency room visits. However, these studies used a retrospective design, whereas an experimental design, such as a randomized controlled trial, is needed to establish causality. Future studies should more closely examine the temporal relationship between the onset and progression of SDB and comorbid conditions relative to hospitalization and other HCRU outcomes. Further investigation into the sex differences between males and females with SDB is also needed to test the hypotheses generated by these findings and to explore potential biological mechanisms. Overall, these findings can pave the way for future endeavors aimed at evaluating the impact of early detection and tailored management approaches in SDB patients to mitigate hospitalization risks and enhance overall public health outcomes.

## Figures and Tables

**Table 1:** Baseline characteristics.

	Analysis 1: 5-Year Matching					Analysis 2: Year 1 Matching				
Demographics	SDB Group (n = 519,818)	Unadjusted Control Group (n = 883,910)	SMD	Adjusted Control Group (n = 888,619)	SMD	SDB Group (n = 193,671)	Unadjusted Control Group (n = 248,848)	SMD	Adjusted Control Group (n = 260,298)	SMD
Age (mean ± std)	46.27 ± 9.35	45.97 ± 9.62	0.03	45.97 ± 9.61	0.03	48.02 ± 8.49	47.88 ± 8.91	0.02	48.03 ± 8.85	−0.00
18–24 y % (n)	2.38% (12,359)	2.46% (21,783)	−0.01	2.48% (22,049)	−0.01	1.28% (2471)	1.59% (3949)	−0.03	1.52% (3960)	−0.02
25–34 y	10.18% (52,897)	10.91% (96,442)	−0.02	10.83% (96,268)	−0.02	6.63% (12,843)	7.08% (17,628)	−0.02	6.90% (17,965)	−0.01
35–44 y	24.84% (129,105)	25.24% (223,120)	−0.01	25.29% (224,745)	−0.01	21.99% (42,580)	21.83% (54,328)	0.00	21.32% (55,495)	0.02
45–54 y	40.34% (209,719)	39.10% (345,614)	0.03	39.23% (348,565)	0.02	43.17% (83,611)	40.99% (102,006)	0.04	41.34% (107,605)	0.04
55–64 y	22.27% (115,738)	22.28% (196,951)	0.00	22.17% (196,992)	0.00	26.94% (52,166)	28.51% (70,937)	−0.04	28.92% (75,273)	−0.04
Female	38.99% (202,664)	43.07% (380,737)	−0.08	42.54% (378,017)	−0.07	35.45% (68,660)	47.55% (118,330)	−0.25	46.47% (120,970)	−0.23
**Medical Conditions**										
Cancer	9.51% (49,449)	9.60% (84,897)	−0.00	9.48% (84,265)	0.00	10.48% (20,300)	12.61% (31,368)	−0.07	12.38% (32,217)	−0.06
Gastrointestinal disease	41.46% (215,534)	38.92% (344,058)	0.05	38.52% (342,332)	0.06	42.71% (82,710)	46.10% (114,708)	−0.07	45.29% (117,899)	−0.05
Infectious Disease	37.29% (193,842)	36.05% (318,613)	0.03	35.65% (316,830)	0.03	37.33% (72,296)	37.44% (93,168)	−0.00	37.64% (97,970)	−0.01
Mental Health	36.59% (190,192)	34.38% (303,881)	0.05	34.08% (302,799)	0.05	35.63% (69,014)	37.86% (94,216)	−0.05	36.65% (95,390)	−0.02
Musculoskeletal	77.78% (404,332)	77.33% (683,498)	0.01	76.64% (680,999)	0.03	79.96% (154,852)	83.24% (207,153)	−0.09	82.60% (214,997)	−0.07
Maternity and Perinatal	2.30% (11,961)	2.54% (22,448)	−0.02	2.55% (22,697)	−0.02	1.70% (3288)	2.43% (6040)	−0.05	2.37% (6159)	−0.05
Neurologic Disease	26.76% (139,101)	24.87% (219,859)	0.04	24.71% (219,542)	0.05	26.41% (51,139)	30.37% (75,564)	−0.09	29.56% (76,945)	−0.07
Other	98.13% (510,072)	98.23% (868,270)	−0.01	98.18% (872,478)	−0.00	98.18% (190,137)	98.89% (246,092)	−0.06	98.76% (257,058)	−0.05
Respiratory	69.66% (362,101)	68.77% (607,821)	0.02	68.11% (605,270)	0.03	69.61% (134,807)	74.33% (184,964)	−0.11	73.40% (191,060)	−0.08
**Cardiometabolic Conditions**										
DM	23.10% (120,090)	14.82% (130,965)	0.22	19.38% (172,170)	0.09	28.13% (54,481)	16.23% (40,394)	0.29	22.49% (58,553)	0.13
AD	13.16% (68,399)	9.93% (87,743)	0.10	10.56% (93,818)	0.08	14.33% (27,759)	12.15% (30,225)	0.07	14.00% (36,435)	0.01
DM or AD	31.35% (162,974)	21.95% (194,050)	0.22	26.46% (235,119)	0.11	36.51% (70,701)	24.90% (61,956)	0.26	31.53% (82,078)	0.11

Note: Table 1 shows baseline characteristics for the sample for each of the two analyses. Note that the sample size is different for each group due to differences in the time period for selecting SDB and matched controls in each analysis. DM = diabetes mellitus; AD = atherosclerotic disease; SMD = standardized mean difference.

**Table 2:** Hospitalization outcomes.

Outcome	SMD	SDB Group	Control Group	OR (95% CI)
**Analysis 1: 5 Year Matching**				
Unadjusted		(n = 519,818)	(n = 883,910)	
DM hospitalization % (n)	0.09	4.19% (21,766)	2.60% (22,976)	1.63 * (1.61–1.67)
AD hospitalization % (n)	0.05	3.48% (18,105)	2.62% (23,130)	1.34 * (1.32–1.37)
DM/AD hospitalization % (n)	0.10	7.19% (37,392)	4.95% (43,778)	1.49 * (1.47–1.51)
Adjusted		(n = 519,818)	(n = 888,619)	
DM hospitalization % (n)	0.05	4.19% (21,766)	3.26% (28,987)	1.30 * (1.27–1.32)
AD hospitalization % (n)	0.04	3.48% (18,105)	2.86% (25,421)	1.23 * (1.20–1.25)
DM/AD hospitalization % (n)	0.06	7.19% (37,392)	5.80% (51,574)	1.26 * (1.24–1.28)
**Analysis 2: Year 1 Matching**				
Unadjusted		(n = 193,671)	(n = 248,848)	
DM hospitalization % (n)	0.10	4.23% (8186)	2.52% (6274)	1.71 * (1.65–1.76)
AD hospitalization % (n)	0.03	2.73% (5288)	2.34% (5821)	1.17 * (1.13–1.22)
DM/AD hospitalization % (n)	0.09	6.55% (12,688)	4.60% (11,442)	1.45 * (1.42–1.49)
Adjusted		(n = 193,671)	(n = 260,298)	
DM hospitalization % (n)	0.04	4.23% (8186)	3.46% (9018)	1.23 * (1.19–1.27)
AD hospitalization % (n)	0.01	2.73% (5288)	2.54% (6620)	1.08 * (1.04–1.12)
DM/AD hospitalization % (n)	0.04	6.55% (12,688)	5.68% (14,774)	1.17 * (1.14–1.19)

Note: n = sample size for each group per analysis. This also represents the denominator for the hospitalization rates. SMD = standardized mean difference; all SMD between −0.10 and 0.10; OR = odds ratio; CI = confidence interval; * odds ratio 95% CI > 1.00.

**Table 3:** Hospitalization outcomes by sex.

	Male			Female		
Outcome	SDB Group	Control Group	OR (95% CI)	SDB Group	Control Group	OR (95% CI)
**Analysis 1: 5 Year Matching**						
Unadjusted	(n = 317,154)	(n = 503,173)		(n = 202,664)	(n = 380,737)	
DM hospitalization	3.92% (12,447)	2.60% (13,078)	1.53 * (1.49–1.57)	4.60% (9319)	2.60% (9898)	1.81 * (1.75–1.86)
AD hospitalization	3.93% (12,456)	3.17% (15,974)	1.25 * (1.22–1.28)	2.79% (5649)	1.88% (7156)	1.50 * (1.44–1.55)
DM/AD hospitalization	7.35% (23,316)	5.46% (27,452)	1.38 * (1.35–1.40)	6.95% (14,076)	4.29% (16,326)	1.67 * (1.63–1.70)
Adjusted	(n = 317,154)	(n = 510,602)		(n = 202,664)	(n = 378,017)	
DM hospitalization	3.92% (12,447)	3.15% (16,070)	1.26 * (1.23–1.29)	4.60% (9319)	3.42% (12,917)	1.36 * (1.33–1.40)
AD hospitalization	3.93% (12,456)	3.44% (17,588)	1.15 * (1.12–1.17)	2.79% (5649)	2.07% (7833)	1.36 * (1.31–1.40)
DM/AD hospitalization	7.35% (23,316)	6.22% (31,771)	1.20 * (1.17–1.22)	6.95% (14,076)	5.24% (19,803)	1.35 * (1.32–1.38)
**Analysis 2: Year 1 Matching**						
Unadjusted	(n = 125,011)	(n = 130,518)		(n = 68,660)	(n = 118,330)	
DM hospitalization	4.00% (4997)	2.69% (3506)	1.51 * (1.44–1.57)	4.64% (3189)	2.34% (2768)	2.03 * (1.93–2.14)
AD hospitalization	3.02% (3771)	3.01% (3926)	1.00 (0.96–1.05)	2.21% (1517)	1.60% (1895)	1.39 * (1.29–1.48)
DM/AD hospitalization	6.61% (8261)	5.36% (6997)	1.25 * (1.21–1.29)	6.45% (4427)	3.76% (4445)	1.77 * (1.69–1.84)
Adjusted	(n = 125,011)	(n = 139,328)		(n = 68,660)	(n = 120,970)	
DM hospitalization	4.00% (4997)	3.63% (5062)	1.10 * (1.06–1.15)	4.64% (3189)	3.27% (3956)	1.44 * (1.37–1.51)
AD hospitalization	3.02% (3771)	3.20% (4464)	0.94 (0.90–0.98)	2.21% (1517)	1.78% (2156)	1.25 * (1.16–1.33)
DM/AD hospitalization	6.61% (8261)	6.43% (8952)	1.03 (1.00–1.06)	6.45% (4427)	4.81% (5822)	1.36 * (1.31–1.42)

Note: n = sample size for each group per analysis. This also represents the denominator for the hospitalization rates. SMD = standardized mean difference; all SMD between −0.1 and 0.1; OR = odds ratio; CI = confidence interval; * odds ratio 95% CI > 1.00.

## Data Availability

Data from this study were used based on an established data use agreement and are available via commercial license from Merative.

## Supplementary Materials

The following supporting information can be downloaded at: https://www.mdpi.com/article/10.3390/jcm13247715/s1, Table S1: Classification schema for medical conditions of interest.
