# Supplementary material for: The Impact of Comorbid Sleep-Disordered Breathing on Hospitalization Risk Related to Diabetes and Atherosclerotic Disease: A Retrospective Cohort Analysis
Source: J Clin Med. 2024 Dec 18;13(24):7715. doi: 10.3390/jcm13247715 (PMC11677575; doi:10.3390/jcm13247715)
Supplement: Supplementary file 1 [file jcm-13-07715-s001.zip › jcm-3269241-supplementary.pdf]

Supplementary Table S1. Classification Schema for Medical Conditions of Interest.

	ICD-10 Codes	CPT-4 Procedure codes	Disease-identifying medications by GPI
Sleep-Disordered Breathing (SDB)	G47.3xx	94660	
Diabetes mellitus (DM)	E08.xxx-E13.xxx, O24.xxx, P70.2xx, T38.3xx, T85.614x, T85.624x, T85.633x, T85.694x, T85.72x, Z46.81x, Z79.4xx, Z96.41x	0141T, 0142T, 0143T, 3044F-3072F, 48155, 48160, 48554, 48556, 80432, 84681	27x, 9720x, 9410003x, 9705103090x, 9705105x
Atherosclerotic disease (AD)			
Cardiac dysrhythmias and abnormalities of heartbeat	I44.xxx, I45.xxx-I49.xxx, P29.1xx, R00.x, Z45.01x, Z45.02x, Z95.0xx, Z95.810	00410, 00530, 00534, 00537, 33200-33261, 80176, 80190-80194, 93228, 93229, 93279-93284, 93288-93299, 93600-93662, 93724, 93745, 93727-93738, 93741-93744	35x
Cerebrovascular disease, hemorrhagic	I60.xxx-I62.xxx, I67.1xx, I69.0xx-I69.2xx		
Cerebrovascular disease, ischemic	G45.xxx, G46.xxx, I63.xxx-I66.9xx, I67.2xx, I67.8xx, I69.3xx, Z86.73x	0075T, 0076T, 35501-35510, 37195, 37215, 37216	8515005x
Congestive Heart Failure (CHF)	I09.8xx, I11.0xx, I13.0xx, I13.2xx, I42.xxx, I50.xxx, I51.5xx, I51.7xx	0001F, 1004F, 4014F	3110x
Coronary artery disease (CAD)	I20.xxx-I25.xxx, Z95.1xx, Z95.5xx, Z98.61x	0009F, 0010F, 1002F, 00566, 00567, 33508, 33510-33572, 35600, 92920-92979	3210x
Peripheral vascular disease (PVD)	I70.xxx-I72.xxx, I77.7xx, I73.9xx, Z98.62x	0078T-0081T, 33860-33877, 35302-35400, 35450-35476, 35506-35587, 35606-35683, 35700-35907, 37201, 37205-37209, 93668	8520x
Chronic kidney disease, requiring dialysis (CRF/ESRD)	N18.6xx, Z49.xxx, Z91.15x, Z99.2xx	36825, 36830, 36830-36838, 36904-36909, 90918-90999	5280x, 9917x, 9970x
Chronic kidney disease, not requiring dialysis (renal insufficiency)	N18.1xx, N18.2xx, N18.3xx, N18.4xx, N18.5xx, N18.9xx, N19		
Acute kidney failure	N17.x, T79.5xxx		
